# Initial psychometric evaluation of the Korean version of the Canine Brief Pain Inventory for canine osteoarthritis

**DOI:** 10.4142/jvs.26077

**Published:** 2026-07-14

**Authors:** Inwoo Park, Byung-Jae Kang

**Affiliations:** 1 Department of Veterinary Clinical Sciences, College of Veterinary Medicine and Research Institute for Veterinary Science, Seoul National University, Seoul 08826, Korea.; 2 BK21 FOUR Future Veterinary Medicine Leading Education and Research Center, Seoul National University, Seoul 08826, Korea.

**Keywords:** Clinical metrology instrument, owner-reported outcome, pain assessment, factor analysis, construct validity

## Abstract

**Importance:**

Accurate assessment of osteoarthritis-related pain in dogs requires validated, language-specific clinical metrology instruments. The absence of a Korean version of the widely used Canine Brief Pain Inventory (CBPI) limits standardized pain assessment and outcome evaluation in Korean-speaking clinical and research settings.

**Objective:**

This study aimed to develop and perform an initial psychometric evaluation of the Korean version of the CBPI.

**Methods:**

This observational cross-sectional study included 56 dogs with osteoarthritis and 22 healthy controls. Construct validity was assessed using exploratory and confirmatory factor analyses. Convergent validity was assessed using Spearman correlations between CBPI scores and the owner-reported quality-of-life item. Discriminant validity was evaluated through group comparisons between dogs with osteoarthritis and healthy controls. Internal consistency was evaluated using Cronbach’s α.

**Results:**

Exploratory analysis identified a dominant general pain factor (eigenvalue = 8.04; 80.4% variance). Confirmatory factor analysis demonstrated superior relative fit for the two-factor model (χ^2^/df = 2.94, comparative fit index [CFI] = 0.917, root mean square error of approximation [RMSEA] = 0.186, standardized root mean square residual = 0.044) compared with the one-factor model (χ^2^/df = 4.13, CFI = 0.861, RMSEA = 0.236). CBPI scores showed significant negative correlations with quality of life in dogs with osteoarthritis (ρ = −0.516 to −0.485; corresponding 95% confidence intervals ranged from −0.710 to −0.248; *p* < 0.001). Dogs with osteoarthritis had higher CBPI total scores than healthy controls (median, 18.0 vs. 0; *p* < 0.001). Cronbach’s α coefficients ranged from 0.951 to 0.968.

**Conclusions and Relevance:**

The Korean CBPI demonstrated excellent internal consistency and preliminary evidence of construct validity for assessing osteoarthritis-related pain in dogs, supporting its use as an owner-reported outcome measure in Korean-speaking clinical and research settings. Future longitudinal studies should evaluate its test-retest reliability and responsiveness to treatment.

## INTRODUCTION

Osteoarthritis (OA) is the most common joint disease in dogs and a leading cause of chronic pain, impaired mobility, and reduced quality of life [1]. Pain is a hallmark clinical feature of canine OA. However, its assessment remains challenging in routine veterinary practice, because pain-related behaviors and functional limitations are often subtle, variable, and not fully observable during clinical examinations alone [2]. Consequently, relying solely on veterinarian-based assessments may underestimate the true impact of OA-associated pain in affected dogs [3].

To address these limitations, clinical metrology instruments (CMIs), particularly owner-reported outcome measures, have become essential for assessing and monitoring chronic pain and functional impairment of dogs with OA [4]. CMIs capture multidimensional aspects of pain and function in the home environment, providing clinically meaningful information that complements physical examination findings. The Canine Brief Pain Inventory (CBPI) is one of the most widely used owner-reported outcome measures for assessing pain associated with canine OA [5,6]. As the long-term management of canine OA increasingly relies on sustained analgesic therapy and owner-based monitoring, availability of a validated Korean-language CMI is important for accurate pain assessment and outcome monitoring [7].

The CBPI consists of two related domains: Pain Severity Score (PSS) comprising four items that assess pain intensity and a Pain Interference Score (PIS) comprising six items that evaluate the impact of pain on daily activities and functional performance. All items were scored on a 0–10 numerical rating scale, with higher scores indicating worse pain or greater functional interference. In addition, the CBPI includes a single global quality-of-life (QOL) item [3]. This has been extensively adopted in clinical practice and major randomized clinical trials of OA therapeutics as an owner-reported outcome measure for canine OA [8,9,10].

The linguistic translation of the CMI alone does not guarantee measurement equivalence across cultures. Differences in language and cultural contexts may influence the interpretation and understanding of questionnaire items, which may alter the underlying psychometric structure of the instrument. Without formal validation, translated questionnaires may yield biased or non-comparable results, which may compromise their validity, reliability, and interpretability in both clinical practice and research settings [11,12].

Accordingly, regulatory and methodological guidance emphasizes the importance of validating translated outcome measures before their use in clinical research [13,14,15]. Validation ensures that a translated instrument demonstrates appropriate structural validity, construct validity, and reliability and the results are interpretable across clinical settings and over time [16,17].

Despite the widespread use of the CBPI in canine OA research and clinical practice, a formally validated Korean version suitable for assessing OA-related pain in dogs has not yet been established. Therefore, the objectives of this study were to (a) translate the CBPI into Korean and culturally adapt it using a standardized methodology and (b) perform an initial psychometric evaluation of the Korean version using a population of dogs with OA, including healthy controls for discriminant validation. We hypothesized that the Korean version would demonstrate construct validity and reliability consistent with previously validated versions, and that the original two-factor framework would be supported or provide a theoretically meaningful empirical approximation.

## METHODS

### Translation and cultural adaptation of the CBPI

Permission to translate and use the CBPI was obtained from the original author (Dr. Dottie Cimino Brown) before initiating the study. The translation and cultural adaptation processes were conducted following established international guidelines for the translation and cultural adaptation of outcome measures [11,15].

The translation process consisted of five sequential steps. First, forward translation from English to Korean was performed by a bilingual veterinarian with clinical and research experience in veterinary orthopedics. Second, the translated version was reviewed by three independent bilingual veterinarians to assess conceptual equivalence, linguistic clarity, and clinical relevance. Third, a consensus discussion involving all four reviewers was conducted to resolve any discrepancies and finalize the reconciled Korean version. Fourth, cognitive debriefing was conducted by pretesting five Korean-speaking dog owners to assess item comprehension, wording clarity, ease of completion, and practical comprehensibility among the intended respondents. Formal back translation was not performed. Semantic and conceptual equivalence with the original CBPI was instead assessed through independent review by bilingual veterinarians, consensus reconciliation, and owner pretesting. This process evaluated whether the Korean items preserved the core concepts of the original CBPI, including the temporal distinctions among pain severity items, pain-related interference with daily activities, the 0–10 response anchors, and the global QOL item. Based on feedback from this pretesting, minor revisions were made as necessary. As the final step, the questionnaire was revised, proofread, and finalized. The original English and final Korean versions of the CBPI are provided as Supplementary Data 1 and 2, respectively.

### Study design and population

This study was designed as an observational, cross-sectional study and conducted at the Seoul National University Veterinary Medical Teaching Hospital between May and November 2025. Dogs were prospectively recruited during the study period, and data were collected at the time of enrollment. The target sample size for dogs with OA was determined based on the number of items included in the CBPI. Given that the CBPI consists of 10 scored items, a minimum recommended subject-to-item ratio of 5:1 was applied [18]. This necessitated a sample size of at least 50 dogs for the psychometric analysis. An additional 10% was added to account for potential incomplete responses or participant withdrawal, yielding a target sample size of 55 dogs with OA.

Client-owned dogs were allocated to one of the two groups. The OA group consisted of dogs diagnosed with OA based on compatible clinical and radiographic findings. The control group had no history of orthopedic disease and no clinical or radiographic evidence of OA. Neuter status was not used as an inclusion or exclusion criterion for either group. Dogs were excluded if they had other diseases affecting gait or mobility, had undergone orthopedic surgery within the preceding six months, had incomplete CBPI responses, or their owners were unable to complete the Korean version of the CBPI.

Dog owners completed the Korean version of the CBPI. The demographic and clinical information collected included age, breed, body weight, body condition score, sex, and current use of analgesics.

### Statistical analysis

Descriptive statistics were used to summarize the demographic and questionnaire data and are reported as median (minimum–maximum) for continuous variables and frequency (percentage) for categorical variables. Group comparisons of the OA and control dogs were performed using the Mann-Whitney U test for continuous variables and the χ^2^ test or Fisher’s exact test for categorical variables, as appropriate. Cases with missing values were excluded prior to analysis; therefore, no missing data were present in the final dataset.

Prior to exploratory factor analysis (EFA), the sampling adequacy and factorability of the correlation matrix were evaluated using the Kaiser-Meyer-Olkin (KMO) measure and Bartlett’s test of sphericity. The EFA was performed using maximum likelihood extraction with varimax rotation. Varimax rotation was selected to facilitate interpretability and maintain consistency with prior CBPI validation studies, and correlations between factors were subsequently examined using confirmatory factor analysis (CFA). The number of factors retained was determined based on Kaiser’s criterion (eigenvalues > 1) and a visual inspection of the scree plot. Factor loadings of ≥ 0.40 were considered salient. Communalities (*h*^2^) were examined to assess the proportion of variance for each item explained by the extracted factors.

Based on the theoretical two-factor structure of the CBPI, both the original two-factor model and an alternative one-factor model were evaluated using CFA for comparative purposes. Model fit was assessed using the chi-squared to degrees of freedom ratio (χ^2^/df), comparative fit index (CFI), Tucker-Lewis index (TLI), root mean square error of approximation (RMSEA), and standardized root mean square residual (SRMR). RMSEA was reported with a 90% confidence interval (CI), consistent with conventional reporting in structural equation modeling. The following were considered indicative of acceptable model fit based on commonly used guidelines: χ^2^/df < 3.0, CFI and TLI ≥ 0.90, RMSEA ≤ 0.08, and SRMR ≤ 0.08 [19,20]. The evaluation of model fit and measurement properties followed established psychometric validation frameworks [16,17].

Convergent validity was assessed by examining Spearman’s rank correlation coefficients between CBPI scores and the global QOL item. Bootstrap 95% CIs were calculated for Spearman’s correlation coefficients using 10,000 resamples. Discriminant validity was evaluated by comparing the CBPI scores of the OA and control groups using the Mann-Whitney *U* test. For item-level comparisons of the ten CBPI items between groups, Bonferroni correction was applied to account for multiple comparisons. Composite scores, including PSS, PIS, and CBPI total score, were considered the primary outcomes for discriminant validity. Internal consistency reliability was assessed using Cronbach’s α coefficients and item–total correlations for the PSS, PIS, and CBPI total score. Floor effects were assessed as the proportion of dogs achieving the minimum possible score for each item. All statistical analyses were performed using R software (version 4.5.1; R Foundation for Statistical Computing, Austria). A significance level of 0.05 was used for all hypothesis tests.

## RESULTS

### Study population and descriptive statistics

Seventy-eight dogs, comprising 56 with OA and 22 healthy controls, were included in the study. The demographic characteristics of the study population are summarized in Table 1. There were no statistically significant differences between the OA and control groups in terms of age, body weight, body condition score, or sex distribution. As expected, the proportion of dogs receiving analgesic treatment was higher for the OA than for the control group (53.6% vs. 0%, *p* < 0.001).

**Table 1:** Demographic characteristics of dogs with OA and controls

Variables		OA group (n = 56)	Control group (n = 22)	*p* value
Age (yr)		8.2 (1.1–16.4)	6.9 (1.4–13.2)	0.233
Body weight (kg)		5.7 (2.1–43.6)	6.8 (1.3–29.8)	0.545
Body condition score		6 (4–9)	5 (3–7)	0.292
Sex				0.309
	Female	3 (5.3%)	0 (0%)	
	Spayed female	20 (35.7%)	10 (45.5%)	
	Male	5 (8.9%)	0 (0%)	
	Castrated male	28 (50.0%)	12 (54.5%)	
Analgesic treatment		30 (53.6%)	0 (0%)	< 0.001^***^

Values are presented as number (%) or median (range).

OA, osteoarthritis.

^***^p < 0.001.

### CBPI item scores and floor effects

The median CBPI item scores and floor effects for dogs with OA and controls are provided in Table 2. The median scores for individual pain severity and pain interference items ranged from 1 to 2 for the OA group, whereas the median score for all CBPI items was 0 for the control group.

**Table 2:** CBPI item scores and floor effects in dogs with OA and controls

CBPI item	OA group (n = 56)		Control group (n = 22)	
	Median (min–max)	Floor effect (%)	Median (min–max)	Floor effect (%)
Pain at its worst	2 (0–9)	25.0	0 (0–1)	90.9
Pain at its least	1 (0–7)	48.2	0 (0–0)	100.0
Pain on average	2 (0–8)	28.6	0 (0–0)	100.0
Pain right now	2 (0–7)	28.6	0 (0–0)	100.0
General activity	2 (0–10)	28.6	0 (0–1)	95.5
Enjoyment of life	1 (0–8)	28.6	0 (0–0)	100.0
Ability to rise	2 (0–10)	30.4	0 (0–0)	100.0
Ability to walk	2 (0–9)	28.6	0 (0–0)	100.0
Ability to run	2 (0–10)	26.8	0 (0–1)	90.9
Ability to climb	2 (0–10)	26.8	0 (0–1)	95.5
PSS	6 (0–30)	21.4	0 (0–1)	90.9
PIS	13 (0–57)	12.5	0 (0–2)	86.4
CBPI total	18 (0–87)	10.7	0 (0–2)	81.8

*p* values for individual item comparisons were adjusted using Bonferroni correction.

CBPI, Canine Brief Pain Inventory; OA, osteoarthritis; PSS, Pain Severity Score; PIS, Pain Interference Score.

Floor effects were observed for the OA group, with the highest floor effect noted for the item “pain at its least” (48.2%). In contrast, pronounced floor effects were observed for the control group, with floor percentages exceeding 90% for most CBPI items. For the composite scores, floor effects were 21.4% for the PSS, 12% for the PIS, and 10.7% for the CBPI total score for the OA group, whereas the corresponding values for the control group exceeded 80%.

### Construct validity

#### Sampling adequacy and factorability

The KMO measure of sampling adequacy was 0.90, indicating that the data were suitable for factor analysis. Bartlett’s test of sphericity was significant (χ^2^ = 757.66, df = 45, *p* < 0.001), confirming sufficient inter-item correlations for factor extraction.

#### EFA

An EFA was conducted for the CBPI item scores of dogs with OA (n = 56). Visual inspection of the scree plot and Kaiser’s criterion indicated that only the first factor exceeded an eigenvalue of 1 (eigenvalue = 8.04), accounting for 80.4% of total variance. The second factor had an eigenvalue of 0.58 and explained an additional 5.8% of the variance, suggesting a dominant first factor.

Nevertheless, both one- and two-factor solutions were further examined based on the theoretical two-factor structure of the CBPI. The factor loadings and communalities of both models are presented in Table 3. For the one-factor model, all items had high factor loadings (range: 0.80–0.96) and strong communality (range: 0.64–0.92). For the two-factor model, pain severity items (items 1–4) loaded primarily on factor 1, whereas pain interference items (items 5–10) loaded primarily on factor 2. All factor loadings exceeded the predefined threshold of 0.40, and communalities remained high across items (range 0.61–0.99). The two-factor solution explained a cumulative 81.8% of the total variance.

**Table 3:** Exploratory factor analysis of the Korean CBPI for dogs with osteoarthritis (n = 56)

CBPI item	Factor loading			Communality (*h*^2^)	
	One-factor model	Two-factor model		One-factor model	Two-factor model
		Factor 1	Factor 2		
Pain at its worst	0.93	0.80	0.50	0.87	0.89
Pain at its least	0.82	0.67	0.46	0.67	0.66
Pain on average	0.96	0.87	0.47	0.92	0.99
Pain right now	0.94	0.80	0.50	0.89	0.90
General activity	0.88	0.56	0.70	0.77	0.80
Enjoyment of life	0.80	0.59	0.52	0.64	0.61
Ability to rise	0.89	0.65	0.61	0.80	0.90
Ability to walk	0.90	0.48	0.85	0.81	0.96
Ability to run	0.87	0.49	0.77	0.76	0.84
Ability to climb	0.82	0.49	0.71	0.67	0.74

Factor 1 represents pain severity (items 1–4), and factor 2 represents pain interference (items 5–10).

CBPI, Canine Brief Pain Inventory; *h*^2^, communality.

**Fig. 1:**
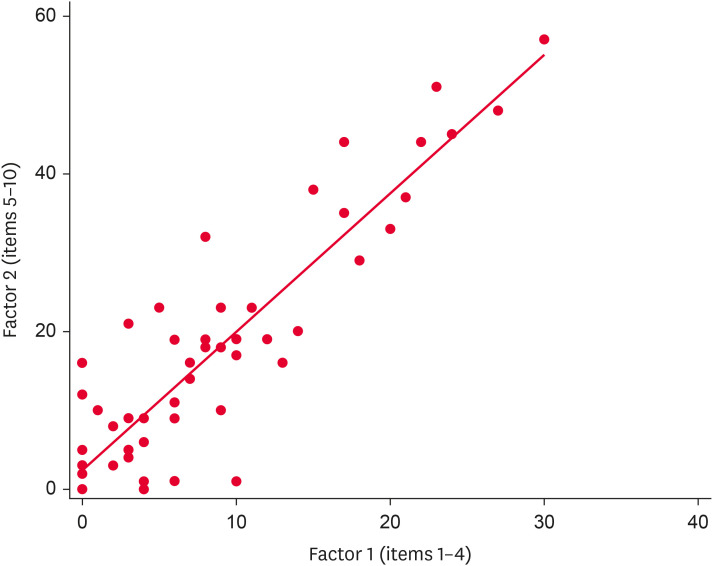
Relationship between Factor 1 (Pain Severity, items 1–4) and Factor 2 (Pain Interference, items 5–10) scores for dogs with osteoarthritis (n = 56). A strong positive Spearman correlation was observed between the two factors (ρ = 0.823, 95% confidence interval, 0.684 to 0.914, *p* < 0.001), indicating that the two factors were highly related. The solid line represents the fitted linear regression line.

A strong positive correlation was observed between Factor 1 (Pain Severity) and Factor 2 (Pain Interference) scores (ρ = 0.823, 95% CI, 0.684 to 0.914, *p* < 0.001), indicating that the two factors were highly related (Fig. 1).

#### CFA

A one-factor model suggested by the EFA and the original two-factor CBPI model were evaluated using CFA for comparison, and the model fit indices are presented in Table 4. The one-factor model demonstrated inadequate fit with a χ^2^/df of 4.13, CFI of 0.861, and TLI of 0.822. The two-factor model showed relatively improved fit with a χ^2^/df of 2.94, CFI of 0.917, TLI of 0.890, and SRMR of 0.044. The RMSEA values exceeded the commonly recommended thresholds for both models, indicating that neither model achieved fully acceptable absolute fit. Nevertheless, the two-factor model demonstrated superior relative fit compared with the one-factor model and was retained for interpretability and theoretical consistency with the original CBPI framework.

**Table 4:** Fit indices for the confirmatory factor models of the Korean CBPI for dogs with osteoarthritis (osteoarthritis, n = 56)

Model	χ^2^	df	χ^2^/df	CFI	TLI	RMSEA (90% CI)	SRMR
One-factor	144.59	35	4.13	0.861	0.822	0.236 (0.197–0.277)	0.046
Two-factor	99.84	34	2.94	0.917	0.890	0.186 (0.144–0.229)	0.044

Fit indices included χ^2^, df, χ^2^/df, CFI, TLI, RMSEA (with 90% CI), and SRMR.

CBPI, Canine Brief Pain Inventory; CFI, comparative fit index; TLI, Tucker–Lewis index; RMSEA, root mean square error of approximation; CI, confidence interval; SRMR, standardized root mean square residual.

### Convergent validity

Convergent validity was assessed by examining the correlation between CBPI scores and the global QOL item for the dogs with OA (*n*=56). Significant negative correlations were observed between QOL and CBPI total score (ρ = −0.507, 95% CI, −0.697 to −0.258, *p* < 0.001), PSS (ρ = −0.485, 95% CI, −0.678 to −0.248, *p* < 0.001), and PIS (ρ = −0.516, 95% CI, −0.710 to −0.260, *p* < 0.001). These findings indicate that higher CBPI scores are associated with lower perceived quality of life, supporting the convergent validity of the Korean CBPI.

### Discriminant validity

Dogs with OA had significantly higher CBPI scores than the healthy controls across all outcome measures (Table 2). The median PSS was 6.0 for the OA group versus 0 for the control group (Mann-Whitney *U* = 145, *p* < 0.001). The median PIS was 13.0 versus 0 (Mann-Whitney *U* = 94, *p* < 0.001), and the median CBPI total score was 18.0 versus 0 (Mann-Whitney *U* = 79, *p* < 0.001). These results support the ability of the Korean CBPI to differentiate between dogs with OA and healthy controls.

### Reliability

The Korean version of the CBPI demonstrated excellent internal consistency. Cronbach’s α coefficients were 0.951 for the PSS, 0.953 for the PIS, and 0.968 for the CBPI total score. The item–total correlations were high across items, ranging from 0.80 to 0.96 for the Pain Severity subscale and from 0.76 to 0.93 for the Pain Interference subscale. This indicated strong internal consistency for each subscale.

## DISCUSSION

The present study provides an initial psychometric evaluation of the Korean version of the CBPI in dogs with OA. Overall, the Korean CBPI demonstrated excellent internal consistency and preliminary evidence of construct, convergent, and discriminant validity. These findings support its potential use as an owner-reported outcome measure for assessing OA-related pain among Korean-speaking dog owners, while test–retest reliability and responsiveness remain to be established.

A pronounced floor effect was observed for the item “*pain at its least*,” with approximately 48% of dogs with OA scoring the minimum value. Similarly, floor effects were observed for several other CBPI items, although they were less marked. The pattern is consistent with findings from prior CBPI validation studies, in which floor effects have been reported in clinically managed OA populations. This may reflect the effects of ongoing pain management and, as suggested in the pain assessment literature, the limited recognition of subtle pain behaviors by owners [21,22,23].

A plausible explanation is the relatively high proportion of dogs in the OA group receiving analgesic treatment (53.6%). Effective pain control may attenuate low-grade pain, and owners may underestimate subtle pain manifestations when functional impairment or behavioral changes are not obvious. These factors may reduce the sensitivity of some CBPI items for detecting low-intensity pain, especially in treated patients [22].

Despite these item-level floor effects, the floor effects for composite scores were substantially lower, indicating that the overall scale remains informative for capturing clinically relevant pain severity and pain-related functional interference.

The EFA indicated a largely unidimensional structure with a single dominant factor explaining the majority of the variance. A two-factor solution corresponding to Pain Severity and Pain Interference was not clearly favored over a one-factor model based on eigenvalues, factor loadings, or communalities alone. This pattern likely reflects a strong conceptual and statistical overlap between pain intensity and pain-related functional impairment in dogs with OA, an observation that has been reported in several validation studies [22,24,25].

Several items in the present analysis—particularly those assessing general activity and enjoyment of life—demonstrated cross-loadings across factors, suggesting that these items captured a broader global pain burden than the purely distinct domains of pain severity or functional interference. Similar findings have been reported for other language validations of the CBPI, including the Swedish and Danish versions. Item-level cross-loadings and minor alternative factor groupings for these versions were interpreted to reflect a conceptual overlap between pain perception and functional impact while largely preserving the overall two-factor framework [21,22].

Nevertheless, the strong inter-factor correlation observed in the current study (ρ = 0.823, 95% CI, 0.684 to 0.914) indicates that pain severity and pain interference were highly related. However, the distinction between the two domains should not be inferred from the correlation magnitude alone. Rather, the two-factor model demonstrated superior fit compared with the one-factor model based on the likelihood-ratio comparison (Δχ^2^ = 44.75, Δdf = 1, *p* < 0.001), together with its theoretical consistency with the original CBPI framework [24]. Therefore, the Pain Severity and Pain Interference domains were retained as closely related but theoretically meaningful subscales. It should be noted that the absolute model fit indices were suboptimal, and neither model achieved fully acceptable absolute fit according to conventional thresholds. Although RMSEA values should be interpreted with caution because this index may overestimate model misfit in studies with relatively small samples or limited degrees of freedom [26], the two-factor model was retained on the basis of superior relative fit and theoretical consistency with the original CBPI framework, rather than on the basis of absolute fit indices.

Taken together, these findings suggest that the Korean CBPI primarily reflects a dominant general pain-related burden, while also providing relative and theoretically consistent support for the original two-subscale framework reflecting pain severity and pain-related functional interference. This interpretation is consistent with the original CBPI framework and aligns with prior validation studies reporting high inter-factor correlations and conceptual overlap between these domains.

The convergent validity of the Korean CBPI was supported by significant negative correlations between CBPI scores and owner-reported quality of life. Higher CBPI scores were consistently associated with poorer perceived quality of life, indicating that the instrument captures clinically meaningful aspects of pain-related burden in dogs with OA. This pattern is consistent with the original CBPI validation and subsequent language adaptations, in which pain severity and interference scores were inversely correlated with global QOL assessments [24,27,28].

Discriminant validity was further demonstrated by clear differences in CBPI scores between dogs with OA and healthy controls. All CBPI composite scores were significantly higher for the OA group, confirming the ability of the instrument to distinguish between populations with and without the disease. A similar extreme group validation approach was applied to the original CBPI development study, in which dogs with OA had significantly higher scores than clinically normal dogs [24]. A comparable discriminative performance has also been reported in subsequent language validations, including Swedish and Danish versions, further supporting the cross-cultural robustness of the instrument [21,22].

The Korean CBPI demonstrated excellent internal consistency, with Cronbach’s α values exceeding 0.95 for both subscales and the total score. The item–total correlations were uniformly high, indicating strong coherence among the items within each domain. Although Cronbach’s α values greater than 0.95 may, in some contexts, raise concerns regarding potential item redundancy, similarly high internal consistency coefficients have been consistently reported for the original CBPI and several validated language adaptations, including the Italian and Portuguese versions [4,24,27]. These findings suggest that the high internal consistency observed in the present study likely reflects the structural coherence of the construct, rather than problematic redundancy. Variability in Cronbach’s α across studies may reflect differences in sample heterogeneity and score variance. Nevertheless, high internal consistency estimates across linguistic and cultural contexts support the coherence of the CBPI item structure, although temporal stability requires test–retest reliability assessment.

This study had some limitations. First, it was conducted at a single referral veterinary hospital, which may limit the generalizability of the findings to broader primary care settings. Second, the overall sample size was modest, which may have affected the stability of the factor analytic solutions and precision of the model fit estimates. Additionally, exploratory and confirmatory factor analyses were conducted using the same sample. Although independent validation samples or split-sample approaches are preferable for confirming factor structure, the modest OA sample size in the present initial study made data splitting inappropriate because it would have resulted in very small subsamples and potentially unstable factor solutions and model fit estimates. Therefore, the CFA results should not be interpreted as definitive confirmation of the factor structure. Rather, CFA was used to compare the original two-factor CBPI framework with a one-factor alternative suggested by the exploratory findings. Replication of the factor structure in larger independent cohorts would provide stronger evidence of structural validity. Third, formal back translation was not included in the translation and cultural adaptation process. Although this may limit the strength of the linguistic validation evidence, semantic and conceptual equivalence was assessed through independent review by bilingual veterinarians, consensus reconciliation, and pretesting with five Korean-speaking dog owners. The review focused on preserving the item-level concepts of the original CBPI, including pain severity, pain-related functional interference, response scale anchors, and the global QOL item. Future studies may further strengthen the cross-cultural validation of the Korean CBPI by incorporating independent back translation and structured cognitive interviews with a larger sample of dog owners. Fourth, test–retest reliability was not assessed; therefore, the temporal stability of the Korean CBPI remains to be established. Finally, responsiveness to clinical changes was not examined. Because these measurement properties were not evaluated, the present findings should be interpreted as preliminary and should not be considered evidence of temporal stability or sensitivity to treatment-related change. Longitudinal studies are warranted to evaluate test–retest reliability and responsiveness to clinically meaningful changes.

Future studies should include larger, multicenter cohorts to evaluate the stability of the factor structure and improve generalizability. Assessment of test–retest reliability and responsiveness to clinical improvement would further strengthen the psychometric evidence for the Korean CBPI.

In conclusion, the Korean version of the CBPI demonstrated excellent internal consistency and preliminary evidence of construct validity for assessing OA-related pain in dogs. EFA suggested a dominant general pain factor, whereas CFA provided relative support for the original two-factor framework comprising Pain Severity and Pain Interference. These findings indicate that the Korean CBPI may be interpreted primarily as a global pain-related outcome measure, with theoretically meaningful support for the original two-subscale framework. Collectively, these results provide preliminary evidence supporting the use of the Korean CBPI as an owner-reported outcome measure in clinical practice and research settings. Further large-scale longitudinal studies are warranted to confirm structural stability and evaluate test–retest reliability and responsiveness.

## SUPPLEMENTARY MATERIALS

Supplementary Data 1 Original English version of the Canine Brief Pain Inventory

Supplementary Data 2 Final Korean version of the Canine Brief Pain Inventory
