# Supplementary material for: Initial psychometric evaluation of the Korean version of the Canine Brief Pain Inventory for canine osteoarthritis
Source: J Vet Sci. 2026 Jul 14;27(4):e63. doi: 10.4142/jvs.26077 (PMC13454806; doi:10.4142/jvs.26077)
Supplement: Supplementary file 1 [file jvs-27-e63-s001.doc]

**Supplementary Data 1.** Original English version of the Canine Brief Pain Inventory

This supplementary file provides the original English version of the Canine Brief Pain Inventory used as the source instrument for translation.

# Description of pain:

Rate your dog's pain:

1. Fill in the oval next to the one number that best describes the pain at its **worst** in the last 7days.

 0 1 2 3 4 5 6 7 8 9 10

No pain	Extreme pain

2. Fill in the oval next to the one number that best describes the pain at its **least** in the last 7 days

 0 1 2 3 4 5 6 7 8 9 10

No pain	Extreme pain

3. Fill in the oval next to the one number that best describes the pain at its **average** in the last 7 days.

 0 1 2 3 4 5 6 7 8 9 10

No pain	Extreme pain

4. Fill in the oval next to the one number that best describes the pain as it is **right now**.

 0 1 2 3 4 5 6 7 8 9 10

No pain	Extreme pain

# Description of function:

Fill in the oval next to the one number that best describes how during the last 7 days **pain has interfered** with your dog's:

## 5. General Activity

 0 1 2 3 4 5 6 7 8 9 10

Does not interfere	Completely interferes

## 6. Enjoyment of Life

 0 1 2 3 4 5 6 7 8 9 10

Does not interfere	Completely interferes

## 7. Ability to Rise to Standing From Lying Down

 0 1 2 3 4 5 6 7 8 9 10

Does not interfere	Completely interferes

8. **Ability to Walk**

| 0  Does not interfere | 1 | 2  | 3  | 4  | 5  | 6  | 7  | 8  | 9  | 10  Completely interferes  |
| --- | --- | --- | --- | --- | --- | --- | --- | --- | --- | --- |
| 9. **Ability to Run** |  |  |  |  |  |  |  |  |  |  |
|  0  | 1 |  2 |  3 |  4 |  5 |  6 |  7 |  8 |  9 |  10 |
| Does not interfere |  |  |  |  |  |  |  |  |  | Completely interferes |

10. **Ability to Climb Stairs, Curbs, Doorsteps, etc.**

 0	1	2	3	4	5	6	7	8	9	10

Does not interfere	Completely interferes

# Overall impression:

11. Fill in the oval next to the one number that best describes your dog's overall quality of life over the last 7 days.

Poor	Fair	Good

Very Good

Excellent
