# Supplementary material for: Initial psychometric evaluation of the Korean version of the Canine Brief Pain Inventory for canine osteoarthritis
Source: J Vet Sci. 2026 Jul 14;27(4):e63. doi: 10.4142/jvs.26077 (PMC13454806; doi:10.4142/jvs.26077)
Supplement: Supplementary file 2 [file jvs-27-e63-s002.doc]

**Supplementary Data 2.** Final Korean version of the Canine Brief Pain Inventory

This supplementary file provides the final Korean version of the Canine Brief Pain Inventory used in this study. The Korean version was developed through forward translation, independent bilingual veterinarian review, consensus reconciliation, and pretesting with Korean-speaking dog owners, as described in the Methods section.

# 통증 정도 평가:

반려견의 통증 정도를 평가해 주세요:

1. 지난 7일 동안 **가장 통증이 심했을 때**의 통증 정도를 선택해 주세요.

 0 1 2 3 4 5 6 7 8 9 10

통증 없음	극심한 통증

2. 지난 7일 동안 **가장 통증이 약했을 때**의 통증 정도를 선택해 주세요.

 0 1 2 3 4 5 6 7 8 9 10

통증 없음	극심한 통증

3. 지난 7일 동안 **평균적인** 통증 정도를 선택해 주세요.

 0 1 2 3 4 5 6 7 8 9 10

통증 없음	극심한 통증

4. **지금** 반려견이 느끼는 통증 정도를 선택해 주세요.

 0 1 2 3 4 5 6 7 8 9 10

통증 없음	극심한 통증

# 생활 기능 평가:

지난 7일 동안 통증이 반려견의 생활에 얼마나 영향을 미쳤는지 평가해 주세요:

## 5.  전반적인 활동성

 0 1 2 3 4 5 6 7 8 9 10

전혀 영향 없음	완전히 방해함

## 6.  생활의 만족도

 0 1 2 3 4 5 6 7 8 9 10

전혀 영향 없음	완전히 방해함

## 7.  누워 있다 일어서기

 0 1 2 3 4 5 6 7 8 9 10

전혀 영향 없음	완전히 방해함

8. **걷기**

| 0  전혀 영향 없음 | 1 | 2  | 3  | 4  | 5  | 6  | 7  | 8  | 9  | 10  완전히 방해함  |
| --- | --- | --- | --- | --- | --- | --- | --- | --- | --- | --- |
| **9. 달리기** |  |  |  |  |  |  |  |  |  |  |
|  0  | 1 |  2 |  3 |  4 |  5 |  6 |  7 |  8 |  9 |  10 |
| 전혀 영향 없음 |  |  |  |  |  |  |  |  |  | 완전히 방해함 |

10. **계단 오르기, 턱이나 문지방 넘기**

 0	1	2	3	4	5	6	7	8	9	10

전혀 영향 없음	완전히 방해함

# 전반적인 삶의 질:

11. 지난 7일 동안 반려견의 전반적인 삶의 질을 평가해 주세요.

나쁨	보통	좋음

매우 좋음

완벽함
